# Heterosis Is Prevalent for Multiple Traits in Diverse Maize Germplasm

**DOI:** 10.1371/journal.pone.0007433

**Published:** 2009-10-13

**Authors:** Sherry A. Flint-Garcia, Edward S. Buckler, Peter Tiffin, Elhan Ersoz, Nathan M. Springer

**Affiliations:** 1 USDA-ARS, Plant Genetics Research Unit, and Division of Plant Sciences, University of Missouri, Columbia, Missouri, United States of America; 2 USDA-ARS, U.S. Plant, Soil and Nutrition Research Unit, and Institute for Genomic Diversity and Department of Plant Breeding, Cornell University, Ithaca, New York, United States of America; 3 Department of Plant Biology, University of Minnesota, St. Paul, Minnesota, United States of America; East Carolina University, United States of America

## Abstract

**Background:**

Heterosis describes the superior phenotypes observed in hybrids relative to their inbred parents. Maize is a model system for studying heterosis due to the high levels of yield heterosis and commercial use of hybrids.

**Methods:**

The inbred lines from an association mapping panel were crossed to a common inbred line, B73, to generate nearly 300 hybrid genotypes. Heterosis was evaluated for seventeen phenotypic traits in multiple environments. The majority of hybrids exhibit better-parent heterosis in most of the hybrids measured. Correlations between the levels of heterosis for different traits were generally weak, suggesting that the genetic basis of heterosis is trait-dependent.

**Conclusions:**

The ability to predict heterosis levels using inbred phenotype or genetic distance between the parents varied for the different traits. For some traits it is possible to explain a significant proportion of the heterosis variation using linear modeling while other traits are more difficult to predict.

## Introduction

Heterosis, or hybrid vigor, is the increased performance of hybrid progeny compared to their inbred parents [1]–[2]. Heterosis is manifested in increased size, growth rate, and other parameters in the F_1_ generation in crosses between inbred lines [3]–[4], and exploitation of heterosis is largely responsible for the tremendous increase in maize yield in the United States between the1930's and the 1970's [5]. Despite the importance of heterosis, the molecular basis of this phenomenon is unclear [6]–[7].

Twentieth century corn breeders have spent vast resources developing inbred lines that, when tested in hybrid combinations, produce high yielding hybrids [5], [8]. Hybrid testing programs, both private and public, are expensive and limited in the number of hybrids that can be generated and tested each year. Thus, the ability to predict hybrid performance without producing hybrid progeny or conducting field trials would be valuable, and heterotic groups have been established to facilitate breeding efforts [8]–[9]. New inbreds are developed by selection of germplasm within one heterotic group. These new inbreds are then tested by crossing to an inbred line from an opposite heterotic group. The use of heterotic groups in breeding limits the potential allelic combinations possible in a particular hybrid but does provide for more efficient testing of new hybrids. Attempts to predict the degree of heterosis using heterotic groups or genetic distance between parents as predictor variables have, however, been of limited success [10]–[11].

One reason that robust predictors of heterosis have been difficult to develop may be that most of the focus has been on predicting heterosis for yield. This focus is understandable given that yield is the trait of primary importance in maize breeding. Nevertheless, the focus on yield – arguably the most genetically complex and integrative trait of maize and all other plants – may have hindered the advancement of our understanding of the genetic basis of heterosis and the development of predictors of heterosis. It is clear that heterosis is also expressed for phenotypic traits other than yield (examples provided in references [4], [12]–[15]).

In this study we examine the relationship between heterosis for multiple phenotypic traits and the genetic distance between parents, using data from a large set of diverse inbred lines and the hybrids formed by crossing them to the inbreds B73 or Mo17. Analysis of data for multiple traits in many lines provides an opportunity to make inferences about the underlying mechanisms of heterosis, and the correlations between heterosis for multiple traits. We use data from a large population grown in three environments to develop predictive equations, then test the power of those equations by using them to predict heterosis for a distinct set of genotypes.

## Results

### Prevalence of heterosis in maize hybrids

To assess the prevalence of heterosis for multiple traits in maize hybrids; and to estimate correlations between heterosis for a variety of traits, we developed and phenotyped two partially overlapping populations of maize hybrids. The first population (hereafter referred to as population 1) was derived by crossing 293 diverse maize inbred lines from an association mapping population ([16] as male plants to B73. These hybrids and the inbred parents were evaluated in four environments; Florida in 2002, and North Carolina, Missouri and Wisconsin in 2003 (Full dataset provided in Supporting information Table S1). A second population (hereafter referred to as population 2) was produced by crossing a subset of the inbreds used to generate population 1 (N = 115) that were suitable for growth in the upper Midwest (relatively few tropical genotypes were represented) to both B73 and Mo17. Phenotypic information for this population was collected in Minnesota in 2006 (Full dataset provided in Supporting information Table S2). Seventeen traits were measured in population 1 (not all traits were measured in each environment and in some cases there were missing data due to poor germination or no ear production). Seven traits were measured in population 2. It should be noted that one limitation of this study is the use of only two tester lines, B73 and Mo17. Our conclusions regarding heterosis could be affected if these lines produce unusual patterns of heterosis.

Better-parent heterosis was detected for the majority of the traits (Table 1); greater than 90% of the hybrids exhibited better-parent heterosis for 10 of the 17 traits measured in population 1; only two traits, tassel branch count and stem puncture resistance, exhibited better-parent heterosis in fewer than 50% of hybrids. We focused on better-parent heterosis as this is the economically relevant trait, but we also observed mid-parent heterosis in the majority of hybrids for each of these traits. The levels of better-parent heterosis varied widely from an average of 5% for cob diameter (hybrids had cobs with a diameter 5% greater than the parent with the widest cob) to 185% for plant yield (Table 1). Similar trends were noted in the analysis of the seven traits measured in population 2 (Table 2). There was a strong correlation (*r* = 0.87; P = 0.026) in the level of heterosis for the B73 outcross hybrids grown in Minnesota (population 2) compared to the same genotypes measured in multiple environments for the population 1. For all ear traits except cob diameter, we detected higher average heterosis values for the Mo17 outcrosses than the B73 crosses in population 2 (Table 2).

**Table 1:** Heterosis in population 1.

Phenotype	n	Average %BPH	%BPH values^a^	Inb-Hyb correlation (*r*)	GD-BPH^b^ correlation (*r*)
Days to anthesis^c^	1038	13%	100%	0.88**	−0.55**
Plant yield (g/plant)	333	185%	98%	0.06*	0.57**
Tassel length (cm)	474	22%	97%	0.67**	0.28**
Tassel branch count	474	4%	46%	0.69**	−0.01
Tassel angle^c^	474	30%	99%	0.48**	−0.11**
Plant height (cm)	966	21%	84%	0.69**	0.41**
Upper leaf angle^c^	727	19%	98%	0.54**	−0.22**
Leaf width (cm)	904	9%	86%	0.56**	0.14**
Leaf length (cm)	942	12%	93%	0.65**	0.45**
Stem puncture resistance	437	−12%	21%	0.35**	−0.04
Stem width (cm)	217	15%	79%	0.51**	0.09
10 kernel weight (g)	860	24%	86%	0.26**	0.36**
Cob Diameter (cm)	863	5%	71%	0.51**	0.22**
Kernel Height (cm)	863	30%	97%	0.29**	0.45**
Ear Length (cm)	861	30%	96%	0.47**	0.45**
Cob Weight (g)	862	66%	96%	0.34**	0.45**
Total Kernel Weight (g)	861	144%	99%	0.10**	0.56**

Statistical significance is indicated by ** (P<0.01) and * (P<0.05).

a The % BPH values refers to the percent of hybrids that exhibit better-parent heterosis.

b GD-BPH refers to the genetic distance (GD) and better-parent heterosis (BPH).

c For these traits the better-parent value was the lower value.

**Table 2:** Heterosis in population 2.

		B73 outcross hybrids				Mo17 outcross hybrids			
Phenotype	n	Average %BPH	%BPH values^a^	Inb-Hyb correlation (*r*)	GD-BPH^b^ correlation (*r*)	Average %BPH	%BPH values^a^	Inb-Hyb correlation (*r*)	GD-BPH^b^ correlation (*r*)
Cob diameter (cm)	101	−2.6%	29.6%	0.27**	0.18**	−0.5%	37.4%	0.45**	0.05*
Cob weight (g)	101	15.3%	67.0%	0.11**	0.36**	66.6%	80.9%	0.37**	0.14**
Ear length (cm)	102	12.3%	73.9%	0.17**	0.43**	24.2%	83.5%	0.09*	0.29**
Plant height (cm)	112	25.5%	97.4%	0.16**	0.61**	26.0%	97.4%	0.29**	0.29**
Individual kernel weight (g)	101	0.3%	51.3%	0.57**	0.003	0.3%	45.2%	0.54**	0.05*
Total kernel weight (g/ear)	101	50.6%	76.5%	−0.088	0.55**	119.7%	86.1%	0.15**	0.27**
Seed number (per ear)	101	41.2%	75.7%	−0.234	0.55**	94.6%	85.2%	0.15*	0.16**

Statistical significance is indicated by ** (P<0.01) and * (P<0.05).

a The % BPH values refers to the percent of hybrids that exhibit better-parent heterosis.

b GD-BPH refers to the genetic distance (GD) and better-parent heterosis (BPH).

The average level of better-parent heterosis varied widely for the different traits (Tables 1 and 2). The majority of traits exhibited heterosis of 10%–30%. Plant yield and total kernel weight showed the highest levels of heterosis with hybrid phenotypes more than 100% greater than the better-parent in both populations. It has been suggested that plant yield is a multiplicative trait that integrates variation from several other traits and therefore it may be expected that this trait would exhibit higher levels of heterosis [17]–[18]. A small proportion of the traits exhibited <10% average better-parent heterosis. It is interesting that such strong, prevalent heterosis was observed even in wide-crosses with non-adapted genotypes.

The broad- and narrow-sense heritability were calculated for each trait (Supporting information Table S3). It should be noted that relative values for narrow-sense heritability are more useful than the absolute values since our crossing design precluded obtaining reliable estimates of narrow-sense heritability. The relative level of broad-sense heritability for the traits in inbreds was inversely correlated with the average percent better-parent heterosis (*r* = −0.64; P = 0.0056). However, the levels of narrow-sense heritability did not correlate with the levels of better-parent heterosis (*r* = −0.03; P = 0.91).

### Correlation of hybrid and inbred performance

**Figure 1:**
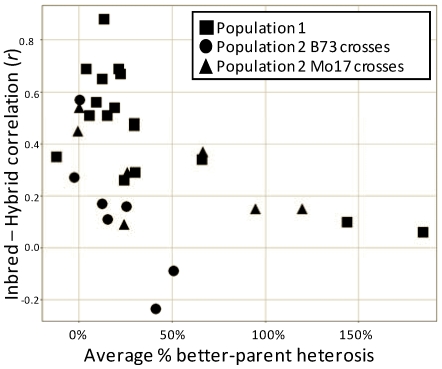
Traits with high levels of heterosis exhibit low correlations between inbred and hybrid phenotypic values. The average level of better-parent heterosis (BPH) is plotted (x-axis) relative to the R correlation value for the inbred and hybrid phenotypic values (y-axis).

Plant breeders are primarily interested in identifying hybrids with superior phenotypic performance. One of the simplest methods for predicting hybrid phenotype might be based on inbred parent phenotype. In our experiment the correlation between the phenotype of the hybrid and the phenotype of the pollen parent (all hybrids were produced with B73 as the seed parent) varied widely among traits varying from *r* = 0.06 for plant yield to *r* = 0.88 for days to tassel (Tables 1 and 2). Interestingly, the traits with greater heterosis (i.e. plant yield and total kernel weight) exhibit relatively weak correlations between inbred and hybrid phenotypes. A plot of the inbred-hybrid correlation relative to the average percent better-parent heterosis for each trait reveals a significant negative trend (Figure 1) suggesting that as the amount of heterosis for a trait increases, the ability to predict the hybrid phenotype based upon the parental phenotype decreases. This may not be particularly surprising – the lower the heterosis the more similar the hybrid trait is to the higher of the two parental values.

### Weak correlations of heterosis for different traits

**Figure 2:**
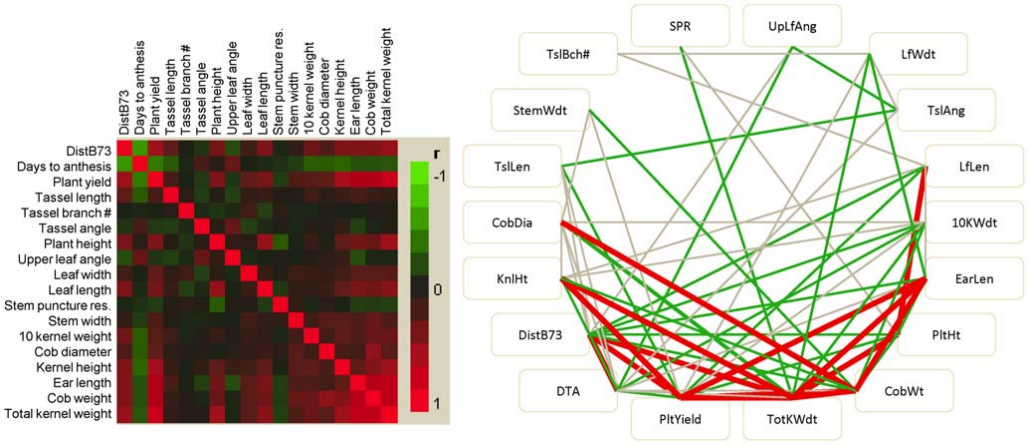
Correlations between better-parent heterosis for 17 phenotypic traits and genetic distance (DistB73) for population 1. (A) The strength and direction of the correlations among the different traits are indicated by the color (red indicates positive correlations while green indicates negative correlations, and the shading represents the strength of the correlation). (B) A correlation network diagram was made to visualize subsets of traits that are highly correlated. All statistically significant correlations are shown by connecting lines. The red lines indicate correlations >0.5, the green lines indicate correlations <0.5 and >0.3 and the gray lines indicate correlations <0.3. Full ontologies for these traits are available in the Methods section.

**Figure 3:**
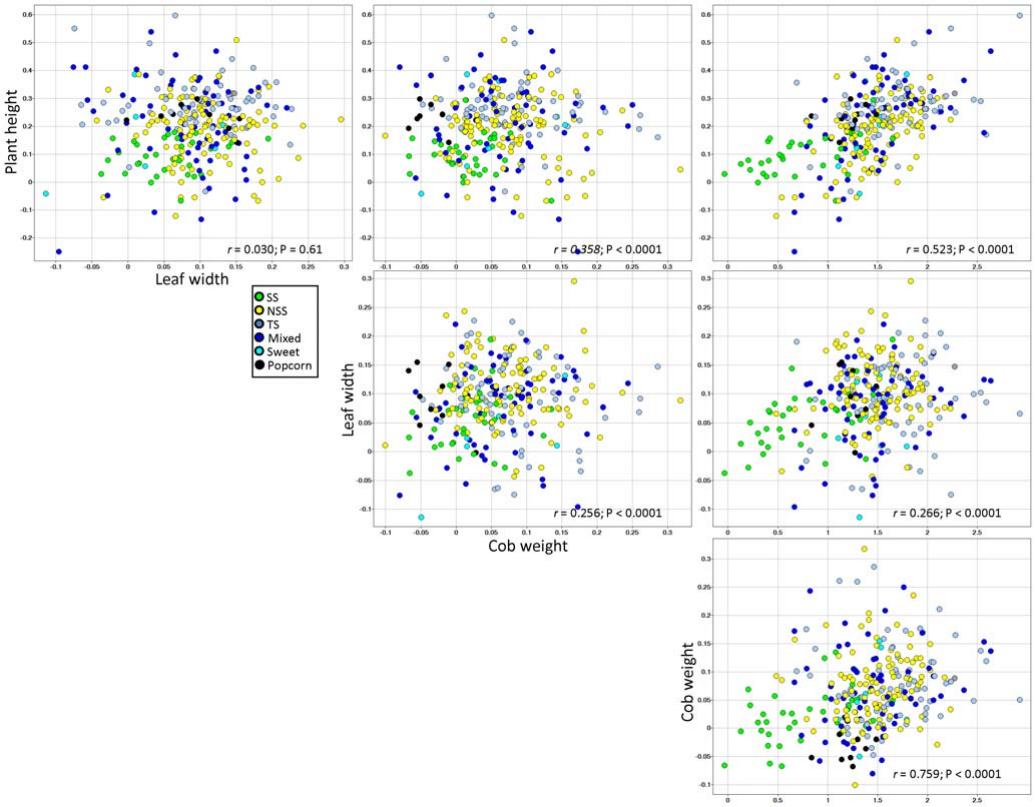
Relationships between better-parent heterosis for plant height, leaf width, cob diameter and total kernel weight in population 1. The color coding indicates the subpopulation of the inbred parent that was crossed to B73.

Heterosis is often treated as a single trait such that specific hybrids are referred to as highly or lowly heterotic. If heterosis is a property of the genotype then the level of heterosis would be correlated for different traits. We found the opposite to be true – the strength of the correlation varied substantially depending upon which traits were being compared (Figures 2 and 3, Table 3). Often, the most significant correlations were observed for traits that likely share a common genetic, physiological, and/or developmental basis such as ear length and total kernel weight, or plant height and leaf length. For traits that are unlikely to have a related basis, such as plant height and leaf width, the correlation of better-parent heterosis levels is quite low (*r* = 0.049). Interestingly, some traits were highly correlated to many other traits, while others were not. Heterosis for traits such as stem width, stem puncture resistance, tassel angle, upper leaf angle and tassel branch count exhibit very few significant correlations with heterosis for other traits (Table 3). By contrast, heterosis for total kernel weight, plant yield, days to anthesis, kernel height, ear length and cob weight were significantly (P<0.05) correlated with heterosis for many (>10/17) other traits (Table 3). This may be a result of the complex nature of certain traits; integrative traits such as kernel weight and plant height would be correlated with other traits while more simple traits such as leaf angle or tassel branch count might not be correlated with other traits.

**Table 3:** Correlations (*r*) for better-parent heterosis among traits for population 1.

Trait	DTA	PltYld	TslLen	TslBr#	TslAng	PltHt	UpLfAng	LfWd	LfLen	SPR	StWd	10KWt	CobDia	KerHt	EarLen	CobWt	TotKWt	Avg R
Days to anthesis		###	###	−0.030	**0.177**	0.041	**0.321**	−0.010	−0.054	−0.088	−0.145	###	###	###	###	###	###	0.226
Plant yield (g/plant)			**0.182**	−0.003	−0.119	**0.455**	−0.102	**0.284**	**0.409**	−0.147	**0.240**	**0.275**	**0.282**	**0.598**	**0.656**	**0.649**	**0.799**	0.366
Tassel length (cm)				0.059	###	0.011	−0.177	0.078	0.129	0.010	0.030	**0.158**	0.061	0.107	**0.195**	0.130	0.079	0.125
Tassel branch count					−0.044	−0.111	−0.051	**0.157**	###	0.036	−0.030	−0.039	0.006	−0.007	−0.011	0.009	0.010	0.046
Tassel angle						0.058	**0.335**	###	−0.054	0.000	−0.026	0.024	0.027	0.085	###	−0.036	0.013	0.095
Plant height (cm)							0.094	0.049	**0.561**	###	−0.013	0.138	−0.006	**0.284**	**0.379**	**0.350**	**0.569**	0.225
Upper leaf angle								−0.043	−0.018	0.028	−0.016	0.024	0.059	0.127	###	−0.037	−0.027	0.112
Leaf width (cm)									0.128	−0.119	0.092	0.096	0.126	**0.287**	**0.401**	**0.376**	**0.458**	0.178
Leaf length (cm)										0.005	0.142	0.117	0.087	**0.174**	**0.274**	**0.277**	**0.247**	0.194
Stem puncture resistance											0.095	−0.029	0.040	−0.002	−0.139	−0.123	###	0.080
Stem width (cm)												0.145	**0.271**	**0.249**	0.217	**0.337**	**0.213**	0.138
10 kernel weight (g)													**0.165**	**0.298**	**0.220**	**0.350**	**0.330**	0.181
Cob Diameter (cm)														**0.211**	0.145	**0.523**	**0.266**	0.163
Kernel Height (cm)															**0.287**	**0.433**	**0.566**	0.265
Ear Length (cm)																**0.671**	**0.724**	0.318
Cob Weight (g)																	**0.727**	0.336
Total Kernel Weight (g)																		0.354
DistB73	###	**0.569**	**0.287**	−0.008	−0.113	**0.414**	###	0.142	**0.453**	−0.039	0.087	**0.359**	**0.222**	**0.446**	**0.452**	**0.445**	**0.556**	0.316

Values in bold are significant at P<0.05.

### Limited correlation of heterosis with genetic distance and heterotic groups

**Figure 4:**
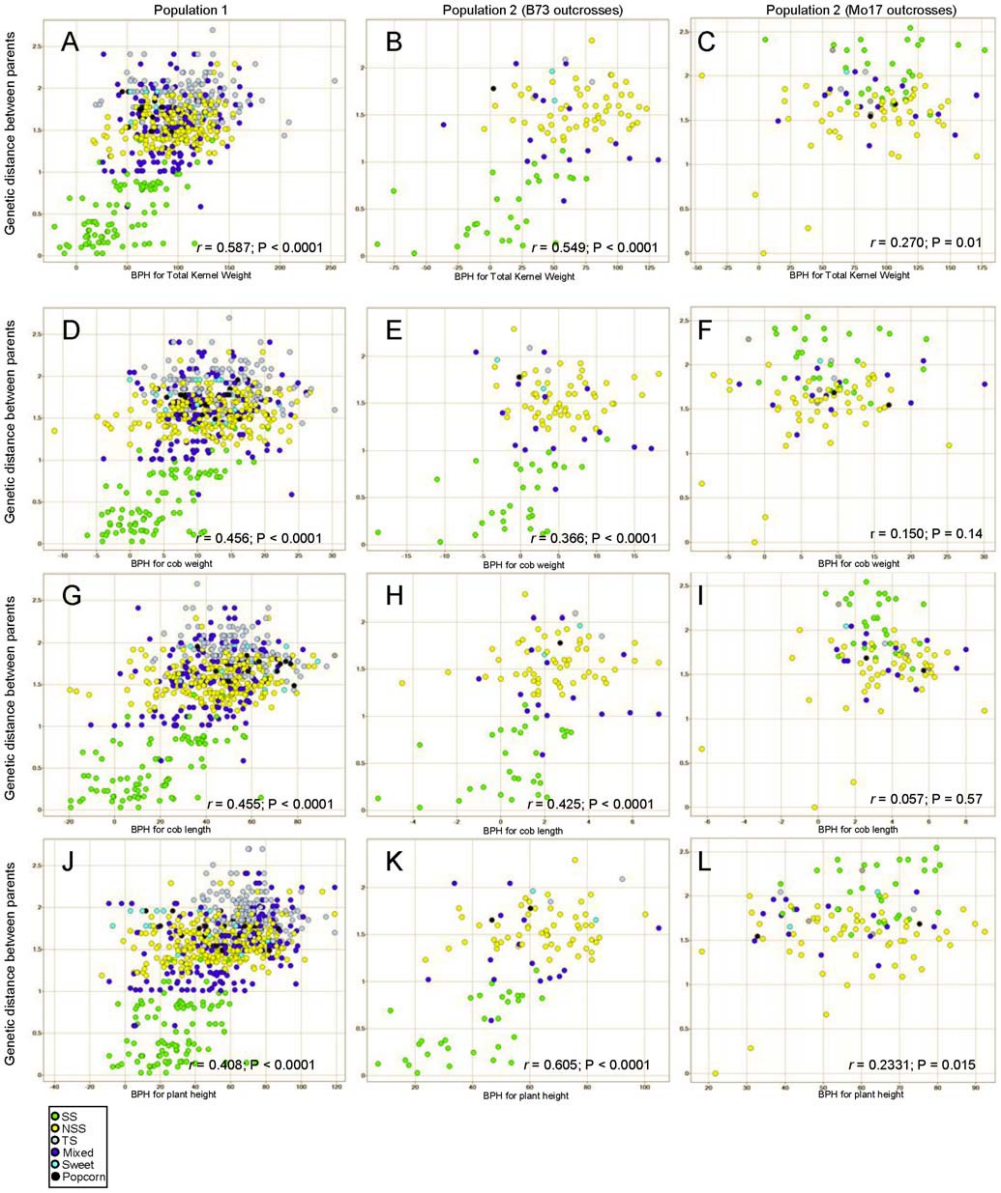
Genetic distance between parents only partially explains heterosis response. The genetic distance between the parents (y-axis) was compared to the better-parent heterosis for total kernel weight (A–C), cob weight (D–F), cob length (G–I), and plant height (J–L). Separate plots were performed for the heterosis values determined in the first population (A, D, G, and J), the B73 outcross hybrids in the second population (B, E, H, and K) and the Mo17 outcross hybrids in the second population (C, F, I, and L). Each data point is color coded to reflect the subpopulation of the non-B73 or non-Mo17 parent (see materials and methods for classification).

**Figure 5:**
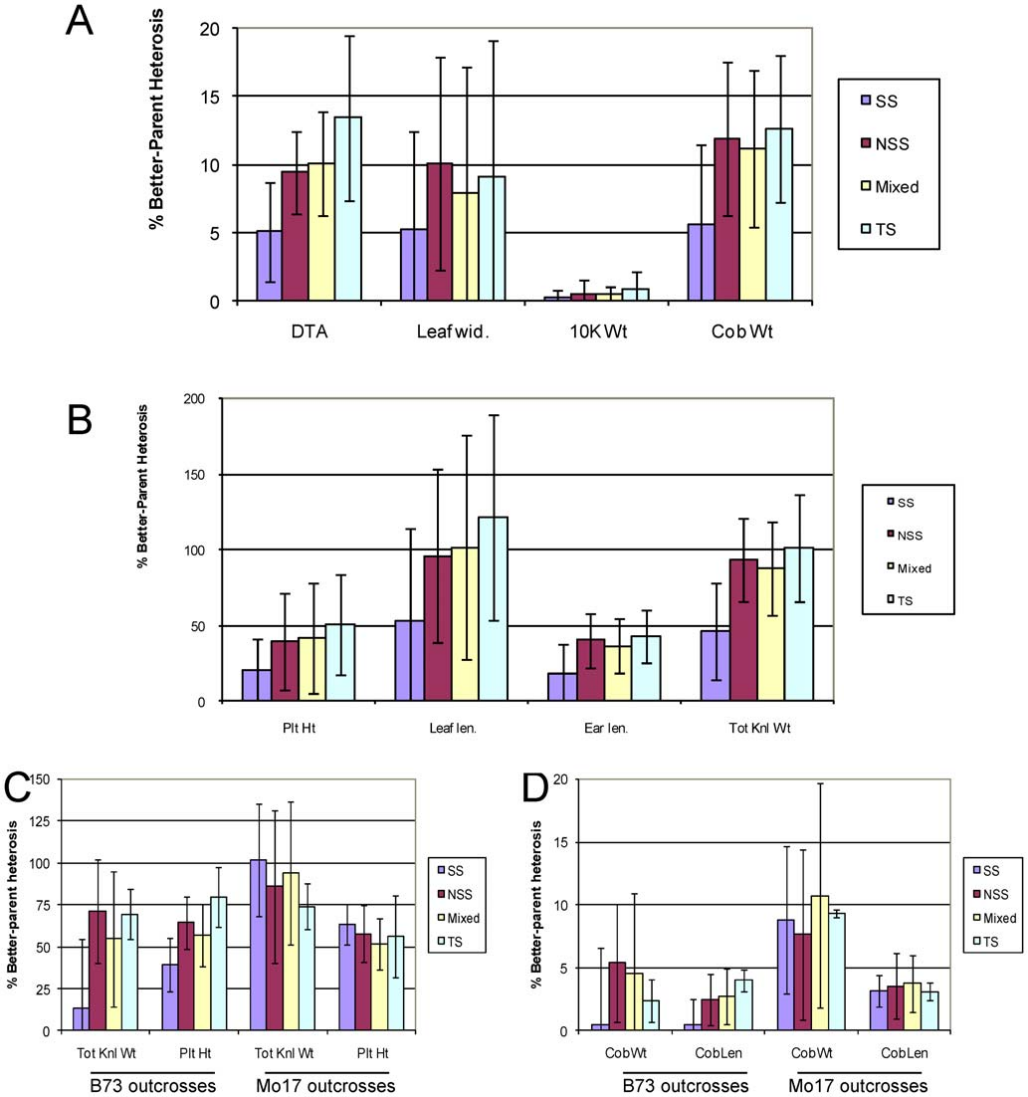
Crosses between heterotic groups increase average better-parent heterosis. The average level of better-parent heterosis was calculated for all hybrids with a parent in the same subpopulation: SS – stiff stalk; NSS – non-stiff stalk; Mixed – mixed parentage; TS – tropical/sub-tropical. A and B (shown as separate plots due to different y-axis scales): The average level of heterosis for hybrids from the same type of cross was determined for days to tassel (DTT), leaf width (LEAFWDT), 10 kernel weight (10 KWt), cob weight (CobWt), plant height (PltHT), leaf length (LEAFLEN), ear length, and total kernel weight (TotKnlWt) traits measured on all 264 hybrids in population 1. C and D (shown as separate plots due to different y-axis scales): The average level of better-parent heterosis was determined for the B73 and Mo17 outcross hybrids in population 2. Note, B73 is a SS while Mo17 is a NSS. The standard deviation for each trait is also shown.

Early research suggested that low genetic diversity will result in low yield heterosis [10] leading some to think genetic distance may be a good predictor of heterosis. While some data support a positive relationship between genetic diversity and heterosis this seems to hold only for closely related inbred lines. Typically the genetic distance between two maize inbred lines is generally a poor predictor of heterosis for yield [11]. Our data support this last statement – the correlation between genetic distance between the two parents and better-parent heterosis for a given trait was often statistically significant but the proportion of variation explained was generally low (Table 1, Figure 4). Very similar trends were observed if we used mid-parent heterosis instead of better-parent heterosis. For traits with genetic distance as a statistically significant predictor of heterosis (noted in Table 1), the correlations appear to be largely due to differences in the response of closely and more distantly related genotypes; closely related genotypes generally show much lower heterosis than more distantly related genotypes (Figures 4 and 5). This suggests that while genetic divergence is required for heterosis, it is a poor predictor of highly heterotic hybrid combinations.

The concept of heterotic groups has been widely used to simplify maize breeding [9]. Generally, inbred lines are divided into different “heterotic groups” and new inbred lines are derived by making crosses within the same heterotic group. To assess hybrid performance, the newly derived inbred lines are then crossed to tester lines from the opposite heterotic group. Genotype information available for the lines used in this study allowed for assignment of each inbred to sub-populations (i.e. heterotic groups) based on genetic markers [16]. We then assessed how the sub-population of the inbred parents affected the average level of heterosis for each of the traits (Figure 5). In population 1 (293 diverse inbreds crossed to B73) the average level of heterosis of stiff stalk (SS) inbreds crossed to B73 (a SS line) was lower than that of inbreds from the other sub-populations (Figure 5A). In population 2 that included crosses by both B73 and Mo17 (a non-stiff stalk (NSS) inbred), there was evidence that crosses within a heterotic group tended to exhibit lower heterosis than crosses between heterotic groups (Figure 5B), although the standard deviations of these values were much larger than the average difference between parents. Thus, while within group crosses tend to exhibit lower heterosis than between group crosses there are many exceptions and group identity appears to be a poor predictor of heterosis.

### Development of models to predict heterosis for four traits

It would be useful to be able to predict hybrid performance and thus heterosis, without actually making crosses and scoring phenotypes. As noted above, correlations between genetic distance and inbred phenotypes and the hybrid phenotypes are generally weak. In addition, it has been proposed that heterosis results from the combination of unique adaptations to new environments [7]–[8]. This would suggest that heterosis might be maximized by identifying hybrids that are derived from crossing two genetically distant lines that have been bred for similar environments. To evaluate this we calculated relative maturity distance which is the difference in relative maturity between the location that the inbred line was developed and the relative maturity of the testing environment for each of our hybrid lines grown in each environment. For four traits exhibiting a range of heterosis – plant height, cob diameter, cob weight, and total kernel weight – we developed predictive linear regression models using phenotype of the inbred parent, genetic distance between parents and relative maturity distance as well as all possible interaction terms (Table 4) as predictor variables. We developed these models using data from population 1, and then applied them to population 2 which differed both in the environment in which plants were evaluated and in the inclusion of both B73 and Mo17 as common parental inbreds.

**Table 4:** Standard least squares model information.

	Cob Weight	Cob diameter	Plant height	Total kernel weight
R-square value (full model)	0.31	0.329	0.701	0.371
Model F-ratio	37.02	40.179	224.16	48.115
Genetic distance F-ratio	174.6	57.2	161.5	232.4
RM distance F-ratio	n.s.*	18.9	268.2	28.8
Inbred phenotype F-ratio	75.4	247.2	146.0	34.2
GenDist*RM Dist F-ratio	n.s.*	n.s.*	n.s.*	n.s.*
Inbred*GenDist F-ratio	7.1	n.s.*	n.s.*	10.1
Inbred*RM Dist F-ratio	6.8	n.s.*	19.5	n.s.*
Inbred*GenDist*RM Dist F-ratio	3.3	n.s.*	n.s.*	n.s.*

* Indicate non-significant values (n. s.). All other values are significant (P<0.05).

**Figure 6:**
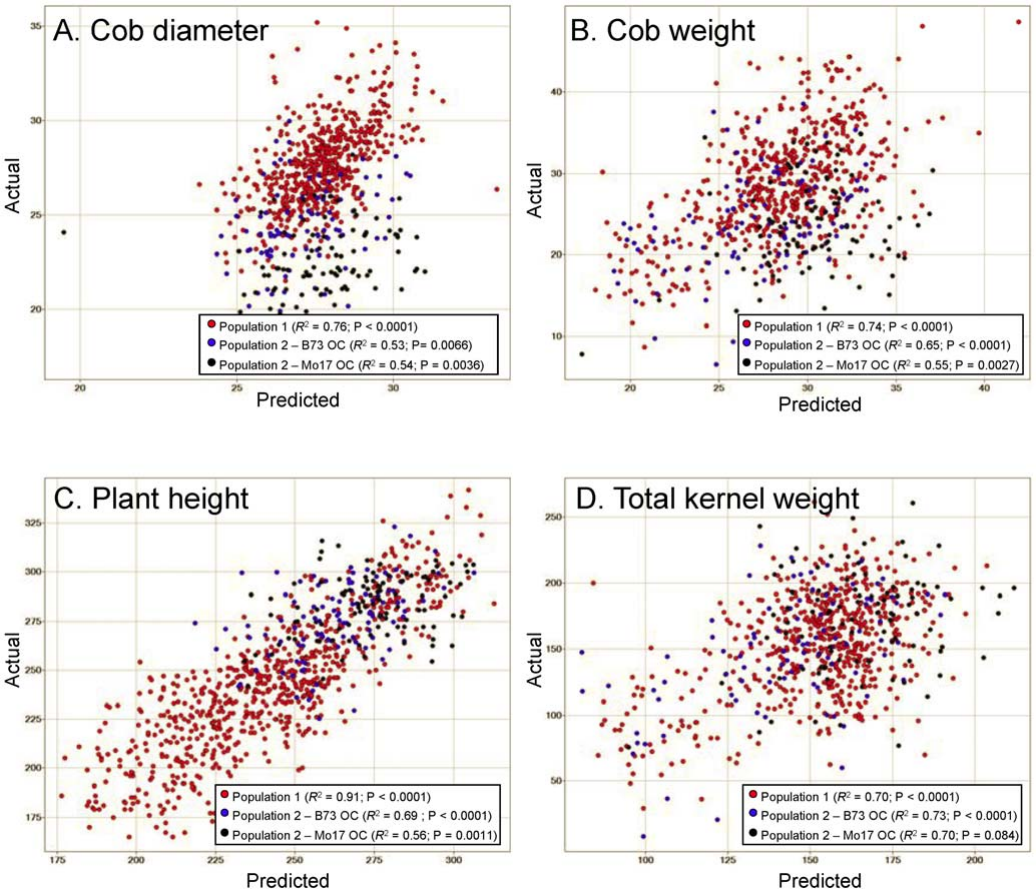
Linear modeling of hybrid performance. A linear model was created using data from population 1 (296 hybrids grown in three summer environments). The linear model included the inbred phenotype, the genetic distance between parents, and the difference between the relative maturity in which the inbred was developed and the relative maturity where the material was grown. This linear model was then used to predict values for population 1, as well as both the B73 outcrosses (B73 OC) and Mo17 outcrosses (Mo17 OC) in population 2 (115 hybrids). The actual hybrid phenotypic values (y-axis) were plotted relative to the predicted hybrid phenotype (x-axis) for cob diameter (A), cob weight (B), plant height (C), and total kernel weight per ear (D). The proportion of variance in actual values explained by the predicted values (R^2^) and P values are shown in the legend for each plot.

ANOVA revealed that the three potential explanatory values (parental phenotype, genetic distance between parents and relative maturity distance) exhibited significant F-ratios for all traits except relative maturity distance in the cob weight model and that the interaction terms were generally not significant (Table 4). The relative F-ratios for the three explanatory values differed for the four hybrid traits. Genetic distance explained the greatest amount of variance in cob weight and total kernel weight, while inbred phenotype explained the greatest amount of variance in cob diameter. The relative maturity distance (the difference in relative maturity between the location that the inbred line was developed and the relative maturity of the testing environment), was significant for three of the four traits and was quite useful in predictions of certain traits such as plant height. Relative maturity distance (a factor that provides a quantitative measurement of the environment) was not significant for the one trait with similar levels of heritability for heterosis in all four environments (Cob Weight). The linear regression model developed from population 1 data in three different environments was a good predictor of heterosis in population 2 for both B73 and Mo17 outcrosses (Figure 6). As expected, the predictive power the model was substantially lower for population 2 than population 1. Nevertheless, when looking at all traits except total kernel weight, there was a significant (P<0.01) correlation between the actual and predicted values of heterosis for both B73 and Mo17 outcrosses in population 2.

## Discussion

### Heterosis is prevalent in maize

The analysis of heterosis for multiple traits in a large set of hybrids reveals that maize hybrids exhibit better-parent heterosis for nearly any trait in nearly every hybrid. Most of the traits measured in this study exhibit better-parent heterosis in over 90% of the tested hybrids. This better-parent heterosis is present for more hybrids, more traits and at considerably higher levels than in Arabidopsis [19]–[21] or in tomato [22]. Semel et al [22] report that better-parent heterosis is observed primarily for reproductive traits related to yield. In this study we have found much higher levels of heterosis for the reproductive traits plant yield and total kernel weight per ear than for other traits. However, we also observed significant better-parent heterosis for a number of traits that would not be considered to be reproductive traits, i.e. leaf angle, leaf width, stem width. It has been suggested that yield is a multiplicative trait that integrates quantitative variation for other traits [17]–[18]. According to this hypothesis, the lower levels of heterosis observed for other traits may interact in a non-linear fashion to produce higher heterosis levels for yield.

Better-parent heterosis was observed for most hybrids even though this study included many hybrid genotypes that would not be evaluated in commercial breeding programs due to the non-adapted nature of tropical germplasm. The crosses of inbred lines from within the same heterotic group (B73 crossed to other stiff-stalk lines or Mo17 crossed to other non-stiff stalk lines) often resulted in heterosis. In most cases these crosses exhibited relatively low levels of better-parent heterosis, but there are examples of high levels of heterosis from crosses of these related lines. On the other end of the spectrum we also evaluated hybrids from crosses between adapted and non-adapted materials (i.e. stiff-stalk by tropical/subtropical hybrids). These hybrids with a large genetic distance between the parental inbreds often do show better-parent heterosis for most of the traits studied.

Previous studies have suggested that genetic distance exhibits a complex relationship with heterosis such that increasing genetic distance between parents results in increasing heterosis but, at high levels of genetic distance the amount of heterosis begins to decline [10]–[11]. We found significant correlations between genetic distance and heterosis for a number of traits. However, we did not find evidence for declining heterosis at large genetic distances. Instead, hybrids with a large genetic distance between parents seemed to exhibit high variance in the amount of heterosis.

### Heterosis is not likely to have a single underlying mechanism

The mechanisms of heterosis have been the subject of intensive research over the past century. Often researchers attempt to identify a single mechanism that might explain the phenomena of heterosis. If this were the case, then we would expect that heterosis for different traits would be highly correlated. However, this is not what we observed in maize hybrids, suggesting that a common measurement for a hybrid, such as genetic distance between parents, will be insufficient to explain heterosis for all traits. This finding corroborates results from QTL analyses which reveal that the genetic basis of heterosis for specific traits is multi-genic [22]–[24] and results that find that the loci underlying variation in heterosis are often trait-specific [22], [25]–[26]. Therefore, although many researchers attempt to describe particular hybrids as being highly or lowly heterotic it appears inappropriate to describe a genotype in terms of heterosis. Rather, heterosis appears to be trait-specific.

The majority of studies on heterosis in maize have focused on studying yield heterosis (or yield component traits). Yield heterosis exhibits some unusual characteristics relative to the other traits including very high levels, correlation with heterosis for many other traits and low levels of predictive ability. These factors probably make yield a very difficult trait for which to predict heterosis levels. The fact that yield heterosis exhibits low, but significant correlations with heterosis for many other traits suggests that yield heterosis reflects cumulative influences of heterosis for many traits. It is quite possible that it would be easier to identify specific loci that contribute to heterosis for traits such as plant height or leaf width. However, the specific molecular mechanisms of these loci may or may not reflect the mechanisms that control heterosis for plant yield. The general trend from genetic and molecular studies on heterosis suggests that heterosis is the result of many loci that have small effects and that interact through a variety of molecular mechanisms.

## Materials and Methods

### Plant Materials and Phenotypic Data

#### Population 1

The association population of 302 diverse maize inbred lines [16] was crossed to B73 in the summer of 2002. From this set of materials, 293 inbreds and their B73-hybrids were grown in adjacent one-row plots in a single replicate, along with replicated B73 plots, in Homestead, FL during the winter of 2002. Adequate seed quantities were available to grow a single replicate of 267 inbreds and their B73-hybrids at each of the following environments during the summer of 2003: Raleigh, NC, Columbia, MO, and Madison, WI. In the 2003 environments, the inbreds and hybrids were grown separately in adjacent blocks, with replicated B73 plots within each block. Phenotypic data were collected for five plants per plot, with some plots having fewer values due to poor germination.

Plant data collected in the field included: flowering time as days to anthesis, plant height (cm), upper leaf angle (at the leaf subtending the flag leaf), leaf length and width (cm), tassel length (cm), tassel branch count and angle; stem puncture resistance (rind penetrometer resistance), a measure of stalk strength; and stem width (cm). An estimate of yield per plant was calculated by harvesting all ears from three to five competitive plants for the NC environment.

Ear and kernel (kernels are referred to as “fruit” in the plant ontology database; http://www.plantontology.org) data were collected from self-pollinated (FL environment) or open-pollinated (2003 environments) ears. Data collected on the ears and kernels (fruits) include: ear and cob diameter (cm), cob mass (g), total kernel mass (g/ear), ear length (cm), and 10-kernel mass. Kernel height was calculated by subtracting cob diameter from ear diameter. MaizeMeister, a PDA and bar-code based phenotyping system, was used to facilitate phenotypic data collection (for more information visit www.maizegenetics.net). All phenotypic data from population 1 used in our analyses are provided in table S1.

#### Population 2

In 2005, a subset of 115 inbred genotypes from the full association mapping panel were used as seed parents in crosses with both B73 and Mo17. These 115 lines include the genotypes suitable for growth in the upper Midwest (relatively few tropical genotypes were represented). The 115 lines plus the 230 hybrid genotypes were planted at the Saint Paul Agricultural Experiment station during the summer of 2006 in one-row plots. Data were collected for six traits including cob diameter (cm), cob weight (g), ear length (cm), individual kernel weight (average g weight determined using 50 kernels), total kernel weight (g)/ear, and seed number. Plant height data were collected from eight plants per genotype at anthesis and ear and kernel data were collected for eight open-pollinated ears for each genotype. All phenotypic data from population 2 used in our analyses are provided in table S2.

### Genetic Distance and Population Structure

Collection and analysis of SSR data used to estimate population structure for these inbred lines was described previously [16], [27]–[28]. Briefly, the software package STRUCTURE (Pritchard et al., 2000) was used to identify three genetic groups (sub-populations) within the 302 inbred line population. These sub-populations correspond to stiff stalk, non-stiff stalk, and tropical/subtropical lines. Each inbred line was assigned to a group when membership probabilities were 0.8 or higher, or to a “mixed” group when membership probabilities were less than 0.8. Because sweet corn and popcorn lines are extraordinarily distinct from all other lines due to the intense genetic isolation that occurred during their development as specialty maize, the structure analysis was first conducted without including sweet corn or popcorn genotypes. The SSR data were also used to calculate the log-transformed, proportion-of-shared-alleles distance between each inbred line and each of B73 and Mo17 (hereafter referred to as “genetic distance,” GD) [16].

### Statistical Analyses

The better-parent heterosis (Hybrid phenotype – Better-parent phenotype) and % better-parent heterosis ((Hybrid phenotype – Better-parent phenotype)/Better-parent phenotype) was calculated for each trait and hybrid. Correlations were calculated and general linear models were analyzed using JMP. For each trait, we examined relationships between inbred and hybrid performance, genetic distance, and better-parent heterosis and % better-parent heterosis.

We developed predictive linear regression models using phenotype of the inbred parent, genetic distance between parents, and relative maturity (RM) distance (difference in relative maturity between the location that the inbred line was developed and the relative maturity of the growing environment) as well as all possible interaction terms as independent variables to explain hybrid phenotype (using JMP). The data from the Florida winter growing season were not included because it was difficult to estimate RM for this environment. Broad-sense heritability, a measure of repeatability across environments, was estimated using PROC MIXED procedure of SAS, as described previously [29].

Narrow sense heritability can be estimated using degree of resemblance between relatives [30]. Estimation of variance components with mixed model using restricted maximum likelihood approaches (REML) and an additive genetic relationship matrix [31] can be utilized to estimate narrow sense heritability since
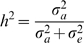
where σ2a and σ2e are variance components directly estimated by REML.

## Supporting Information

Table S1 Phenotypic data for population 1.
(1.16 MB XLS)

Table S2 Phenotypic data for population 2.
(0.10 MB XLS)

Table S3 Heritability estimates.
(0.03 MB XLS)
